# Effects triggered by tumor necrosis factor-α in immortalized murine dental pulp and pre-osteoblastic cells

**DOI:** 10.1590/1807-3107bor-2025.vol39.087

**Published:** 2025-09-08

**Authors:** Giuliana de Campos Chaves LAMARQUE, Roberta Duarte LEME, Luciano Aparecido de ALMEIDA, Marília Pacifico LUCISANO, Karina Fittipaldi BOMBONATO-PRADO, Raquel Assed Bezerra SEGATO, Anne GEORGE, Francisco Wanderley Garcia PAULA-SILVA

**Affiliations:** (a) Universidade de São Paulo – USP, School of Dentistry of Ribeirão Preto, Department of Pediatric Dentistry, Ribeirão Preto, SP, Brazil.; (b) Universidade de São Paulo – USP, School of Dentistry of Ribeirão Preto, Department of Basic and Oral Biology, Ribeirão Preto, SP, Brazil.; (c) University of Illinois, College of Dentistry, Department of Oral Biology, Chicago, IL, United States.

**Keywords:** Tumor Necrosis Factor-alpha, Stem Cells, Dental Pulp, Odontoblasts, Biomineralization

## Abstract

Tumor necrosis factor-alpha (TNF-α) is a cytokine involved in the immune-inflammatory response. It can induce an odontoblastic phenotype and enhance biomineralization in dental pulp mesenchymal stem cells but does not have the same effect on osteoblasts. The reasons for this differential response, despite the shared lineage of these cell types, are not yet clear. This study examined the effects of TNF-α on immortalized mouse dental pulp stem cells (OD-21) and pre-osteoblastic cells (MC3T3-E1). Cells were treated with recombinant TNF-α at concentrations of 1, 10, and 100 ng/mL. Cell viability, proliferation, and migration were assessed using the MTT, CyQUANT, and wound healing assays, respectively. Gene expression was assessed via real-time RT-PCR, and biomineralization was evaluated using alizarin red staining. Statistical analysis was conducted using one-way ANOVA followed by Tukey’s post-hoc test (α = 0.05). TNF-α did not affect cell viability at any concentration (p > 0.05). Proliferation and migration increased after 12 h, with near-complete wound closure by 24 h. TNF-α promoted proliferation and migration in both cell types. OD-21 cells exhibited high levels of Tnfr1 and Runx2 expression and showed biomineralization. In contrast, MC3T3-E1 cells showed high Tnfr2 levels, suppressed Runx2, and inhibited biomineralization. These results highlight how TNF-α influences different cell types from the same lineage in distinct ways.

## Introduction

The immune-inflammatory response of the pulp tissue is coordinated primarily by odontoblasts, which detect injuries and initiate the deposition of reactionary dentin.^1^ In severe injuries, odontoblast cell death might occur, and reparative dentin formation could be performed by stem cells found in the inner dental pulp, which differentiate into odontoblast-like cells.^1^ Undifferentiated mesenchymal cells (stem cells) of the dental pulp are capable of self-renewal, multi-line differentiation, and production of mineralized structures, and they also present anti-inflammatory and immunoregulatory properties.^2^ Osteoblasts, on the other hand, are multipotent mesenchymal cells originating from the bone marrow, which acts in the growth and maintenance of the skeleton.^3^ Several proteins and signaling pathways are regulated at different stages of osteoblast differentiation to ensure their correct function and homeostasis.^4^ During osteogenesis, the recruitment and proliferation of osteoblast precursor cells are crucial, followed by differentiation into osteoblasts that will produce a non-mineralized extracellular matrix, which will be subsequently mineralized.^5^

Several biochemical mediators are released during dental pulp inflammation to stimulate the innate and adaptive immune responses.^6^ Tumor necrosis factor-α (TNF-α) is an upregulated cytokine during inflammation of the pulp tissue that is released predominantly from macrophages.^6^ High levels of TNF-α were detected in exudates from teeth with apical periodontitis and in pulp tissues from teeth with irreversible pulpitis.^7^ TNF-α has an important role in the bone remodeling process and it is directly related to the immune and inflammatory responses, acting on osteoblast differentiation and mineralization.^8^ Studies have shown that TNF-α can also induce cell proliferation, migration, and differentiation with a mineralizing phenotype in dental pulp cell cultures,^9,10^ and recent evidence indicates that dental pulp stem cells in the presence of inflammatory cytokines enhance odontoblast differentiation and collagen matrix formation.^11^ This cellular differentiation proved to be dose-dependent, with induction occurring at low concentrations and inhibition at high concentrations of TNF-α.^9,10^

TNF-α binds to specific cell surface receptors (TNFR1 and TNFR2). These receptors have different biological structures and functions: TNFR1 acts on cytotoxicity, while TNFR2 regulates the inflammatory response and cell proliferation.^12^ TNFR1 is ubiquitously expressed in mammalian cells and it is a functional receptor for osteoblasts and osteoclasts, regulating antimicrobial and pro-inflammatory events.^13^ The binding of TNF-α to TNFR1 forms a complex that activates the NF-κB and p38 MAPK downstream signaling pathways.^14^ TNFR2, on the other hand, is expressed mainly in immune cells and plays an important role in anti-inflammatory events, immune modulation, and neuronal protection.^11^ It also activates the NF-κB pathway, but at a slower rate than TNFR1, and acts on tissue repair by stimulating cell migration and proliferation.^15^ The balance between these two pathways depends on various factors, such as cell type, cell activation, intracellular and/or extracellular environment, and the concentration of inhibitors of pro-apoptotic proteins.^16^

Activation of multiple TNF-α signaling pathways adds complexity to inflammation and reparative response, influencing cell differentiation, proliferation, and apoptosis. However, the molecular mechanisms involved in the differentiation of dental pulp cells and pre-osteoblasts in response to TNF-α remain poorly understood. Therefore, the present study aimed to investigate the effects triggered by TNF-α in mesenchymal cell lineages, including immortalized mouse dental pulp stem cells (OD-21) and pre-osteoblastic cells (MC3T3-E1). Gaining this insight is important to identify how this mediator acts in the pro-inflammatory and reparative responses of these cells.

## Methods

Immortalized undifferentiated mouse dental pulp cells (OD-21) and pre-osteoblasts (MC3T3-E1 – ATCC CRL-2594) were stimulated with TNF-α to investigate the mechanism involved in cell differentiation and biomineralization.^17,18^ For the experiments, the cells were enzymatically dissociated with trypsin-EDTA solution (0.25% trypsin, 1 mmol/L EDTA, Sigma-Aldrich), counted in a Neubauer chamber using the trypan blue staining protocol, and seeded into 96-well plates. Dental pulp cells were cultured overnight in Dulbecco’s modified Eagle’s medium (DMEM), whereas pre-osteoblasts were cultured in minimum essential medium alpha modification (α-MEM), both supplemented with 10% fetal bovine serum, 1% penicillin/streptomycin in a humidified atmosphere of 95% air and 5% CO_2_ at 37 ºC_._

To assess cell viability, cells were seeded at a density of 1 × 10^4^ cells/well and kept in an incubator (37ºC with 95% air and 5% CO_2_) for 12 h. Thereafter, the cultures were stimulated with 1, 10, and 100 ng/mL of TNF-α [R&D Systems, Minneapolis, MN (catalog number: 210-TA)], and dissolved in the medium for experimentation for 24 h. Subsequently, 20 µL of a water-soluble tetrazolium salt (MTT; 3-(4.5-dimethylthiazol-2-yl)-2.5-diphenyltetrazolium bromide, Sigma-Aldrich CO., Catalog number M2128) supplemented with 180 µL of Roswell Park Memorial Institute (RPMI) 1640 (Gibco) was added to each well and the plates were incubated at 37ºC in a humidified atmosphere containing 5% CO_2_ and 95% atmospheric air for 4 h in the dark. After the incubation period, the intracellularly reduced insoluble pigment (formazan) was extracted with DMSO for 30 min and absorbance was measured on a spectrophotometer at a wavelength of 570 nm (SpectraMax Paradigm).^19^

For the proliferation assay, cells were seeded at a density of 1 × 10^4^ cells/well and kept in an incubator (37 ^o^C with 5% CO_2_ and 95% air) for 12 h. After that, the cultures were stimulated with 1, 10, and 100 ng/mL of TNF-α for 12, 24, 36, and 48 h. Thereafter, the medium was removed, the cells were washed with PBS, and the plate was frozen at -80ºC. Cell proliferation was evaluated by a fluorescence assay for nucleic acid detection (CyQUANT™ Cell Proliferation Assay Kit; Thermo Fisher Scientific). The dye used exhibits an intense green fluorescence when bound to cellular nucleic acids, allowing for the measurement of the total amount of DNA. At the time of use, the cells were thawed at room temperature and 200 μL of cell lysis buffer and dye was added to each well. The plate was incubated for 5 min at room temperature and protected from light. Fluorescence was evaluated using a fluorimeter with 485 nm excitation and 530 nm emission filters (SpectraMax Paradigm).

In the scratch wound-healing assay, cells were seeded at a density of 1 × 10^5^ cells/well and incubated for 12 h, as previously described.^20^ A scratch was made across the diameter of the confluent monolayer of each well with the tip of a plastic pipette. Subsequently, the cells were stimulated with culture medium or TNF-α. Cell migration was measured using photographs taken immediately after removal of the cells and at 12, 18, and 24 h after treatment under an inverted microscope at 4 × magnification. The scratch area (mm^2^) was determined using Image J software (NIH, USA), and the percentages of wound closure were calculated as previously described.^21^

For qRT-PCR, cells were seeded at a density of 1 ×10^4^cells/well. Adherent cells were treated with 1, 10, and 100 ng/mL of TNF-α. After 1, 7, and 14 days of treatment, the cells were harvested and the mRNA was extracted using PureLink™ RNA Mini Kit (Invitrogen, Carlsbad, USA), according to the manufacturer’s instructions. The isolated RNA was quantified by NanoDrop^®^ One/One^C^ Microvolume UV-Vis Spectrophotometer (Thermo Fisher Scientific, Waltham, USA). After RNA extraction, the cDNA was synthesized using a High-Capacity cDNA Reverse Transcription Kit (Applied Biosystems, Foster City, USA), from 1 µg of total RNA. Aliquots of cDNA were amplified by qRT-PCR using primers for TNF-α receptor 1 - Tnfrsf1a (Mm00441883-g1), TNF-α receptor 2 - Tnfrsf1b (Mm00441889-m1), and the transcription factors Runx2 (Mm00501584-m1), Gapdh (Mm99999915-g1), and Actb (Mm02619580-g1) were used as the normalization for mRNA.

Amplification was performed under the following conditions: 95 °C for 20 s, 40 cycles of 95°C for 1 s, and a cycle of 60°C for 20 s (Step One Plus, Applied Biosystems, Foster City, USA). The results were analyzed based on the value of the threshold cycle (Ct, cycle threshold). The relative quantification of gene expression was performed using the ΔΔCt method.^22^

A previous study revealed that human dental pulp cells presented a dose-dependent response when stimulated with TNF-α.^9^ A concentration of 10 ng/mL was able to induce higher amounts of dentin sialoprotein and dentin phosphoprotein than 50 ng/mL.^10^ Thus, the concentration of 10 ng/mL of TNF-α was chosen for the biomineralization assay. Cells were seeded into 6-well plates at a density of 2 × 10^5^cells/well. After confluence, the cultures were stimulated with 10 ng/mL of TNF-α and grown in standard (DMEM or α-MEM) or biomineralization culture medium. The biomineralization medium consisted of DMEM or α-MEM supplemented with 10 mM β-glycerophosphate (Sigma), 50 µg/mL of ascorbic acid (Sigma), 5% fetal bovine serum, and 1% antibiotics. Biomineralization culture medium without the addition of TNF-α was used as a positive control. The medium was changed every 3 days and cell culture progression was evaluated by phase-contrast microscopy. The cells were maintained in a mineralizing medium for 21 days. Alizarin Red S solution was added following the previously described protocols.^20^ At the time of staining, the medium was removed from the well, and the monolayer was fixed for 10 min using 70% ethanol and stained with 2% alizarin red solution (pH 4.0) for 5 min at room temperature.

Calcium accumulation was quantified after the release of calcium bound to the dye, achieved by treatment with 100 mM cetylpyridine chloride (Sigma) for 1 h under constant agitation. The absorbance of the released dye was determined on a spectrophotometer at a wavelength of 570 nm (SpectraMax Paradigm).

The experiments were performed in triplicate and the data were analyzed using the GraphPad Prism 9.0 Software (Prism, Chicago, USA). The data were compared using one-way ANOVA followed by Tukey’s post-hoc test. The statistical significance level was set at 5% (α= 0.05).

## Results

**Figure 1:**
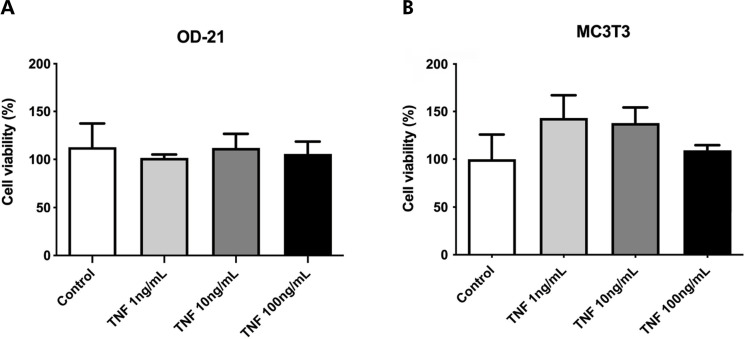
Percentage of cell viability according to MTT assay after 24 hours, in comparison to the untreated group (Control - medium alone); a) Undifferentiated mouse dental pulp cell (OD-21) viability; b) Mouse pre-osteoblast (MC3T3-E1) viability.

**Figure 2:**
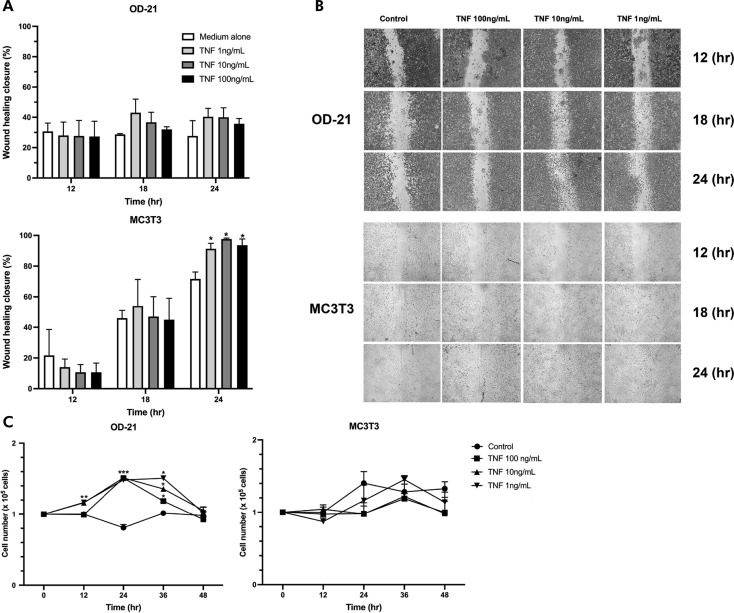
A) Percentage of wound closure after 12, 18, and 24 hours. B) Cell proliferation using a fluorescence quantitative assay after 12, 24, 36, and 48 hours of treatment. C) A wound healing assay was used to evaluate cell migration. *Represent significant statistical differences compared to the control (p < 0.05).

All tested concentrations of TNF-α (1, 10, and 100 ng/mL) did not affect cell viability for dental pulp stem cells or pre-osteoblasts, which demonstrates low or no cytotoxicity (Figure 1). When compared to cell culture media alone, recombinant TNF-α stimulated cell migration and wound closure after 18 h of treatment (Figure 2A). Dental pulp stem cells (OD-21) when stimulated with TNF-α presented cell migration with almost 50% closure after 24 h of treatment but presented no significant statistical differences when compared to the control group (Figure 2A-B). TNF-α also stimulated the migration of pre-osteoblastic cells (MCT3) with significant wound closure (100%) after 24 h at all treatment concentrations (Figure 2A-B). After 12 h of stimulation with 1 and 10 ng/mL of TNF-α, dental pulp stem cells exhibited cell proliferation, and a significant increase in cell number at all concentrations after 24 and 36 h, when compared to the control group (cell culture medium alone). After 48 h, there was no difference in the number of cells that were stimulated by TNF-α in the control group. Although pre-osteoblastic cells presented 100% of wound closure, these cells did not proliferate significantly when compared to the control group in any of evaluated periods (Figure 2C).

**Figure 3:**
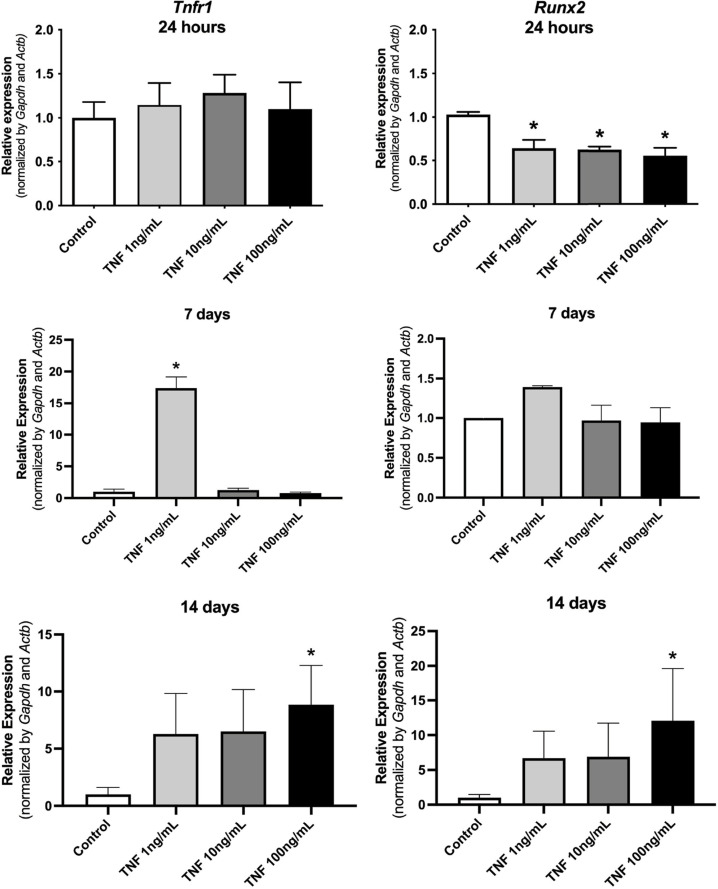
Relative expression of *Tnfrsf1* and *Runx-2* genes, 24 hours, 7 days, and 14 days after stimulation of dental pulp stem cells (OD-21). *p < 0.05 compared to the control.

TNF-α receptor 1 (Tnfrsf1a) expression was significantly higher when treated with a low concentration of TNF-a (1 ng/mL) after 7 days, and at all concentrations evaluated (1,10, and 100 ng/mL) after 14 days of treatment. *Runx2* expression was significantly lower after 24 h of treatment when compared to the control group. However, after 14 days of treatment, *Runx2* expressions were significantly higher for all the TNF-a concentrations evaluated (Figure 3).

**Figure 4:**
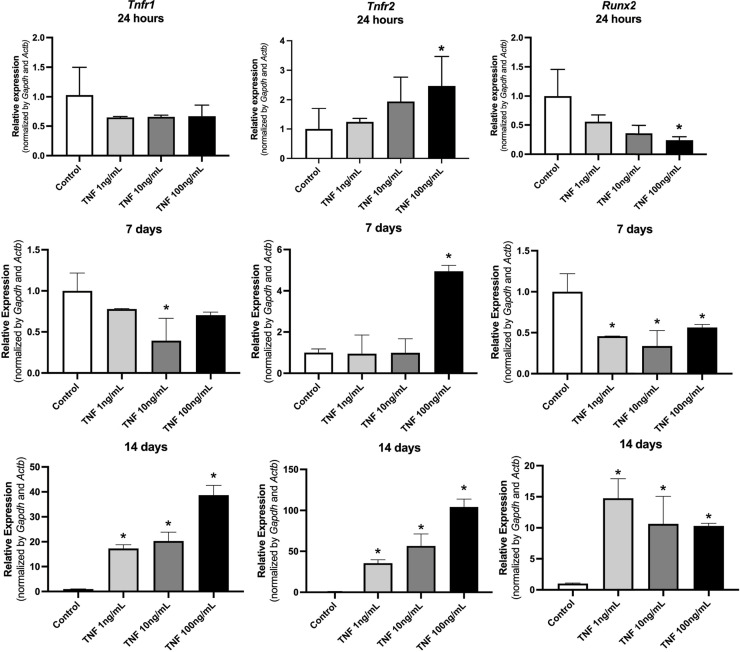
Relative expression of *Tnfr1, Tnfr2, and Runx2* in pre-osteoblasts (MC3T3) after 24 hours, 7 days, and 14 days of treatment. *p < 0.05 compared to the control.

Pre-osteoblasts treated with all concentrations of TNF-α expressed no significant regulation of the TNF-α receptor 1 after 24 h of treatment. After 14 days, however, pre-osteoblasts showed a significant increase in the relative expression of TNF-α receptors 1 and 2 when stimulated with 1, 10, and 100 ng/mL of TNF-α. Cells treated with 100 ng/mL of TNF-α expressed a significant suppression of the expression of transcription factor Runx2 after 24 h and 7 days (Figure 4).

**Figure 5:**
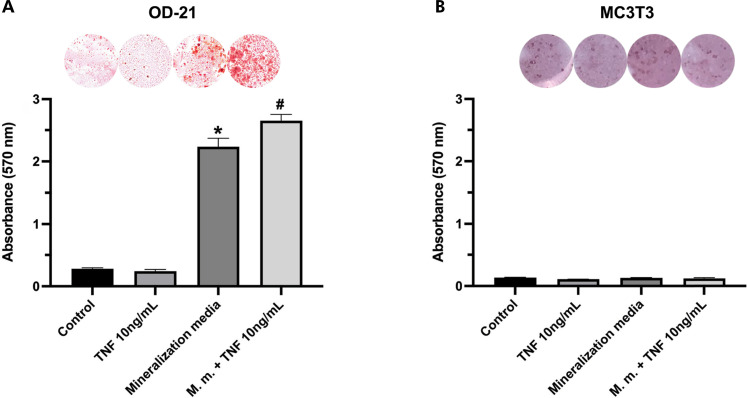
Detection and quantification of the formation of biomineralization nodules after 21 days of stimulation with 10 ng/mL of TNF-α; A) In undifferentiated dental pulp cells (OD-21); B) In pre-osteoblasts (MC3T3). *Represent significant statistical differences compared to the control*.*

OD-21 dental pulp cells showed a significant formation of biomineralization nodules in mineralization media after stimulation with 10 ng/mL of TNF-α for 21 days (Figure 5A). Pre-osteoblastic cells did not form mineralization nodules when stimulated with 10 ng/mL of TNF-α after 21 days (Figure 5B).

## Discussion

Mesenchymal stem cells (MSCs) are considered promising in health science due to their capacity for differentiation, proliferation, and self-renewal.^23^ Studies using DPSCs demonstrated that under pathological conditions and in response to pro-inflammatory cytokines such as TNF-α, these cells differentiate and release dentin matrix proteins.^10^ Regarding osteoblasts, low concentrations of TNF-α were shown to increase osteogenic differentiation.^24^ However, the action of TNF-α and the molecular mechanisms triggered by TNF-α in the dental pulp and pre-osteoblasts are not well established and warrant further investigation.

This study demonstrated that TNF-α was not cytotoxic to the evaluated cells. Previous studies using TNF- α at 0.5 and 10 ng/mL have also shown that, at these concentrations, this cytokine was not toxic to mesenchymal cells.^25^

Dental pulp cells achieved almost 50% wound closure after 24 h, similar to what was previously observed in human periodontal ligament cells, which were not able to close the wound within 48 h.^26^ After 24 and 36 h, an increase in cell number was observed in the groups treated with TNF-α, confirming previous results obtained for human dental pulp stem cells (hDPSCs).^27^ Concentrations of TNF-α ranging from 1 to 200 ng/mL induced hDPSC proliferation in a dose-dependent manner.^27^ In a similar dose-dependent pattern, TNF-α can reduce osteoblast proliferation and induce apoptosis, depending on the stage of cell differentiation.^8^

After 18 h of stimulation, pre-osteoblasts began to migrate and achieved complete wound closure within 24 h. When proliferation was evaluated, pre-osteoblasts did not present a significant increase in cell number. A recent study involving OD-21 cells stimulated with 50 ng/mL of TNF-α also demonstrated the migration ability of these cells with complete wound closure after 48 h, and no proliferation of treated cells when compared to the control group.^28^

In endothelial cells, TNFR1 mediates pro-inflammatory reactions, such as leukocyte activation, and TNFR2 plays a selective role in these reactions, increasing the effects of TNFR1.^29^ In human dental pulp cells, TNFR1 expression has been associated with proliferation and osteogenic differentiation, probably due to activation of the NF-κB pathway.^30^ No significant difference was observed for gene expression of TNFR1 in dental pulp stem cells, and most genes related to an odontoblastic phenotype were not expressed. These findings suggest that genes involved in matrix maturation and mineralization might be activated in a later phase of differentiation in mesenchymal stem cells.^31^

The expression of *Runx2* was also investigated, and our results demonstrate a significant downregulated expression. A similar effect was observed in human periodontal ligament stem cells treated with 1, 10, and 20 ng/mL of TNF-α, indicating that these concentrations may influence the differentiation of stem cells.^32^

One study comparing young to senescent human dental pulp cells found that senescence induces higher levels of TNFR1 when compared to a more youthful state of the cells.^30^ TNF receptor 2 was not detected in OD-21 cells, even when a total mass of 200 ng of total RNA was used for the RT-PCR technique. In fact, in mesenchymal cells, receptor 1 (Tnfrsf1a) is the major TNF-α receptor and it may explain why TNF-α receptor 2 (Tnfr2) was not expressed in this experiment.^15^ Also, soluble TNF-α poorly activates TNFR2, which is commonly used for in vitro studies Efficient activation of this receptor requires the presence of membrane-bound TNF-α or TNF-α treatment with specific ligands.^33^ Also, length of exposure, cell type, and stage of differentiation are factors that can affect the action of TNF-α.^11^ Regarding TNF-α receptors, one study demonstrated that small interfering RNA (siRNA) of TNFR1 reduced mineralization and promoted a downregulation expression of odontoblast-related genes, such as BSP and DSPP in HDPCs,^30^ indicating that this gene can contribute to odontoblast differentiation and mineralization of DPSCs.

Previous reports have shown that TNF-α treatment at a lower concentration (0.1 - 10 ng/mL) increased DPSC mineralization, while a high concentration (50 - 100 ng/mL) suppressed it, indicating a dose-dependent behavior in mineral nodule formation.^30,34,35^ When we evaluated the mineralization potential, DPSC treated with TNF-α presented a significant formation of biomineralization nodules, even though genes related to mineralization had not been detected earlier upon TNF-α stimulation. These findings demonstrate that TNF-α can promote odontoblast differentiation and have a positive effect on the mineralization of DPSCs.

TNF-α acts in bone fracture regeneration, in which short-term exposure to low concentrations was related to the increase in the differentiation of osteoblasts and mesenchymal stem cells.^36^ In pre-osteoblasts (MC3T3-E1), TNFR1 did not increase its expression after stimulation with TNF-α, but TNFR2 did. Very low concentrations of TNF-α (0.5 ng/mL) could significantly enhance TNFR2 expression in MC3T3-E1 cells, and this receptor mediates osteogenic differentiation and bone regeneration.^35,37^ In our study, when higher concentrations of TNF-α were used, pre-osteoblasts expressed high levels of TNFR2 but were unable to differentiate and mineralize. Previous studies have shown that TNFR1 can affect osteoblast differentiation and consequently suppress Runx2 expression, as demonstrated in our study.^25,38^ This suggests that osteogenic differentiation and biomineralization were inhibited in these cells. According to the literature, transcription factor Msx1 is involved in osteoblast differentiation,^39^ and in our study, it was not modulated by TNF-α. Taken together, these findings indicate that TNF-α may interfere with the differentiation of these cells.

Collectively, after 7 days, OD-21 cells exhibited low TNFR1 expression at lower concentrations. By 14 days, Runx2 expression was elevated across all tested concentrations, and biomineralization was detected at 28 days. In MC3T3 cells, a significant increase in Tnfr1 and TNFR2 expression was observed at 14 days, accompanied by a suppression of the transcription factor Runx2 at 7 days, indicating inhibition of osteogenic differentiation and biomineralization in these cells. Regarding genes related to mineralization, alkaline phosphatase was inhibited upon TNF-α stimulation, as demonstrated previously.^25^ In hDPSCs, TNF-α concentrations of 0.1 - 50 ng/mL stimulated DMP1expression and were related to odontoblast differentiation.^40^ Our results show significantly lower levels of this gene, indicating the inhibition of osteogenic differentiation in pre-osteoblasts.

Similar to what occurs with DPSCs, low concentrations of TNF-α (0.1 - 1 ng/mL) promote mineralization and osteogenic differentiation of MC3T3-E1 cells, while concentrations greater than 10 ng/mL inhibit it.^27,37^ In our study, we stimulated cells with 10 ng/mL of TNF-α, and no significant formation of biomineralization nodules was observed. It was demonstrated by several studies that TNF-α activates osteoclastogenesis and decreases bone mineral density by inhibiting osteoblast differentiation.^37^ According to previous reports, the inhibition of mineralization and differentiation of these cells was associated with the suppression of transcription factor Runx2 by TNF-α.^25,38^

Overall, these findings suggest that long-term treatment with the inflammatory mediator TNF-α has distinct effects on osteoblasts and dental pulp stem cells, despite the fact that both cell types originate from the same mesenchymal lineage precursor. Further studies are necessary to expand these results to in vivo experiments to improve the treatment of diseases with mesenchymal stem cells.

## Conclusion

TNF-α showed no toxic effect in osteoblast and dental stem pulp cells and favored cell proliferation and migration. However, osteogenic differentiation and biomineralization were inhibited for pre-osteoblasts. Dental pulp cells, on the other hand, presented a cellular differentiation process, with the production of biomineralization nodules when stimulated by the pro-inflammatory mediator.

## Data Availability

The contents underlying the research text are contained in the manuscript.
